# Synergistic Effects of Multiple Environmental Factors on Degradation of Silicone Rubber Seals under Marine Atmosphere

**DOI:** 10.3390/ma16217013

**Published:** 2023-11-02

**Authors:** Rui-Yuan Wang, Chong-Hao Wang, Ying Wang, Wei-Fang Zhang

**Affiliations:** 1 School of Reliability and Systems Engineering, Beihang University, Beijing 100191, China; wangruiyuan2022@buaa.edu.cn (R.-Y.W.); chonghaowang@buaa.edu.cn (C.-H.W.); ideayichen@163.com (Y.W.); 2 The 41st Institute of Sixth Academy of CASIC, Huhhot 010010, China

**Keywords:** silicone rubber seal ring, degradation, marine atmosphere, multiple environmental factors, accelerating test, reliability, durability

## Abstract

In this research, the degradation behavior and failure mechanism of silicone rubber seal rings under the synergistic effects of multiple factors in the marine atmosphere are fully investigated. Firstly, four aging factors of air, temperature, compressive stress, and chemical medium were determined by analyzing the service environment profile of silicone rubber seal under a marine atmosphere environment. Secondly, to better simulate the actual service environment of silicone rubber and shorten the test period, an artificially accelerated aging test was designed and carried out in the laboratory. In this paper, temperature is utilized as the accelerating stress. According to the results of the pre-test, the accelerating stress level is finally determined to be 110–150 ${}^{\circ }$C. In addition, the compression set applied is consistent with the constant compression permanent deformation value of 28% of the silicone rubber in the actual service process. Finally, through the macroscopic physical properties and microstructure analysis of the samples before and after aging, the corresponding test results are given, and the failure mechanism is analyzed and discussed in detail. Through the above test results and discussion, it can be concluded that the aging process of multi-factor coupling on the lower silicone rubber seal ring is uneven, and its aging process is not a simple superposition of multiple environmental factors. More importantly, the above test data and results are of great significance for evaluating the service life of silicone rubber seals, which can be utilized in the future to improve the reliability and durability of related equipment in the marine environment.

## 1. Introduction

As a common non-metallic material, rubber has a wide range of applications in industrial production and real life, such as insulated wire sheaths [1,2], sealing gaskets [3,4], medical surgical gloves [5], and more [6,7]. Commonly used rubber types are very rich, mainly including natural rubber [8], nitrile rubber [9,10], ethylene propylene rubber [11], silicone rubber [12,13], etc. Compared with other types of rubber, silicone rubber has stronger resistance to heat, ozone, ultraviolet, and other environmental adaptability factors, as well as a better ability to resist compression deformation [14]. Owing to its unique excellent sealing performance, it is often used as a sealing ring in the manufacture of aircraft, ships, and other equipment structures (see references [15,16] and therein).

Despite its excellent properties, such as natural corrosion resistance and resistance to aging, it is undeniable that in the face of the increasingly severe and complex service environments of modern equipment, the long-term exposure to harsh environmental conditions can accelerate the degradation and failure of silicone rubber. To address this issue and enhance its environmental adaptability, durability, and reliability, many experts and scholars both domestically and internationally have conducted pioneering research. In their work, Kaneko et al. [17] conducted a comprehensive study on the degradation mechanisms of silicone rubber under various ultraviolet spectra, laying the groundwork for subsequent research on its ultraviolet aging. Additionally, Liu et al. analyzed the temperature distribution patterns under temperature and pressure loads, which explained the failure of silicone rubber seal structures on barrel hangers in high-pressure and extreme temperature environments, uncovering the reasons for early rubber failure [18]. Considering the aging process of silicone rubber in harsh environments, such as high altitudes and strong salt spray, Zhang et al. conducted a detailed study using ATR-FTIR (Attenuated total reflection-Fourier transform infrared) spectroscopy [19]. Their findings indicated that silicone rubber underwent a degree of degradation, with the main polymer chains breaking, and a reduction in polymerization. Furthermore, there have been numerous notable results in understanding the aging mechanisms of silicone rubber in various other harsh environments [20,21,22].

From the above research, it is evident that although the results have been fruitful and have provided a foundation for silicone rubber degradation research, there are still some drawbacks in terms of methodology and content. Firstly, many degradation tests and data acquisition have relied on field tests in natural environments. While these tests provide accurate results, they come with disadvantages, such as extended testing periods, high costs, and a limited number of data points [23,24]. Secondly, the degradation and failure of rubber in real-world environments is a complex process, and most of the aforementioned studies primarily focus on single environmental factors, with limited exploration of the interactive effects of multiple environmental factors. Finally, the majority of the research concentrated on typical natural environments and industrial atmospheric conditions [25], with relatively few studies on silicone rubber in marine atmospheric environments, resulting in an unclear understanding of its degradation behavior and failure mechanisms. It is important to note that the deficiencies mentioned above are effectively addressed by our experimental work.

To address the research content limitations mentioned earlier, this paper focuses on investigating silicone rubber O-type seal rings utilized in the steering gear of a specific type of missile during service. The study involves the determination of the environmental service profile, which encompasses temperature, compressive stress, and exposure to chemical media. Subsequently, artificially accelerated degradation tests are designed and conducted in a laboratory setting. Post-degradation, the samples’ macroscopic physical properties and microstructure are examined. By combining the degradation behavior of the silicone rubber specimens with changes in their microstructure, this paper delves into the multiple factor degradation mechanism of silicone rubber in marine atmospheric environments.

The main contributions of this paper are primarily reflected in the following three aspects. Firstly, a silicone rubber degradation testing platform was established in the laboratory, and an accelerated testing scheme was designed. This scheme allows for the acquisition of long-term aging results in a natural environment within a short timeframe, resulting in time and cost savings. Secondly, in contrast to the conventional industrial atmosphere and inland environment, this paper comprehensively addresses the degradation behavior and failure mechanisms of silicone rubber in the marine atmosphere. Finally, to better align with the material’s actual service environment, this paper takes into account multiple environmental factors, including temperature, air exposure, compressive stress, and chemical media.

In this article, an accelerated test method in a test chamber was adopted, with temperature as the main accelerating stress factor (110–150 ${}^{\circ }$C), to systematically investigate the coupling between three environmental factors involving temperature, compressive stress and a chemical medium, and their effects on the aging behavior and failure mechanism of silicone rubber seals in the marine atmospheric environment. Meanwhile, comparative tests were carried out under the same aging time and temperature conditions, and the results showed that compressive stress and chemical medium have an important effect on the aging of silicone rubber seals, and multifactorial aging is not a simple superposition of the effects of each environmental factor. Subsequently, by using micro-analysis tools such as AIR-FTIR, X-ray photoelectron spectroscopy (XPS), and scanning electron microscopy (SEM), we investigated the changes in micro-properties of the specimens before and after aging. Meanwhile, by measuring the mass loss rate and compression deformation of rubber before and after aging, we explored the changes in its physical properties. Finally, by combining the results of macro- and micro-analyses, we conclude how multiple environmental factors affect the aging process of silicone rubber in the marine atmospheric environment and provide a corresponding failure mechanism analysis.

## 2. Experimental

In this section, we introduce the silicone rubber materials used in the test and describe the artificial accelerated degradation test methods, including the test scheme and design process, the macroscopic physical properties characterization method, and the microstructure testing and analysis method.

### 2.1. Materials

**Figure 1:**
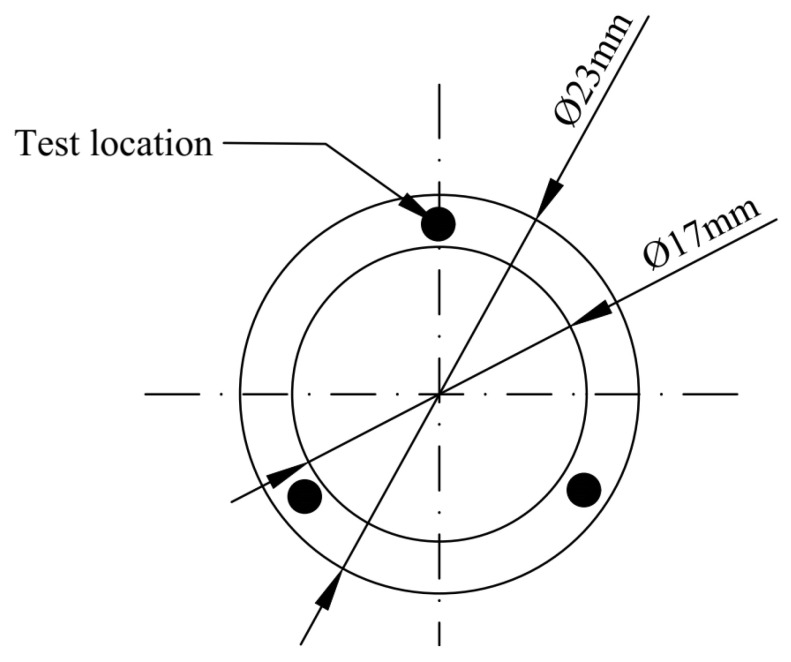
A diagram of the silicone rubber O-ring sample.

The silicone rubber used in this experiment was provided by Aerospace Science and Technology Holding Group Co., Ltd., Harbin, China. The detailed formulations are shown in Table 1. It is a high molecular weight poly-siloxane compound synthesized by introducing vinyl on the basis of siloxane. The materials were processed into O rings that were measured (inner diameter cross-sectional diameter), which is shown in Figure 1. And the chemical medium used in this paper is the Great Wall No. 7017-1 high- and low-temperature grease produced by Henan Aviation Materials Technology Co., LTD., Zhengzhou, China, as is shown in Table 2. The main parameters of the materials are listed as follows.

### 2.2. Analysis of Service Environment Profile of Silicone Rubber

Silicone rubber is often subjected to a variety of environmental factors in the specific use of the environment and aging. Therefore, the profile analysis of the actual service environment of silicone rubber is an important premise to obtain its aging mechanism and improve its reliability.

It is well known to us that compared with the industrial atmospheric environment and inland environment, the marine atmospheric environment has more severe natural environment characteristics, such as high temperature, high humidity, and high salt spray [26,27]. In this paper, the use of silicone rubber O-rings in the steering gear of a certain type of missile in the marine atmosphere environment is taken as the object. The analysis shows that the service environment profile is *temperature*, *air*, *compressive stress*, and *chemical medium*. The compression set is 28%, and the chemical medium is high- and low-temperature grease. Based on the above analysis, this paper studies the degradation process and failure mechanism of silicone rubber under the action of these multiple environmental factors.

### 2.3. Aging Methods

Firstly, in order to study the degradation mechanism and synergism of silicone rubber under high temperature, compressive stress, and chemical medium, in this section, we take the temperature as the accelerated stress. According to the pre-test results, the accelerated stress levels are determined to be 110 ${}^{\circ }$C, 120 ${}^{\circ }$C, 130 ${}^{\circ }$C, 140 ${}^{\circ }$C and 150 ${}^{\circ }$C, and the tests under different working conditions are carried out in the air-circulating oven.

**Figure 2:**
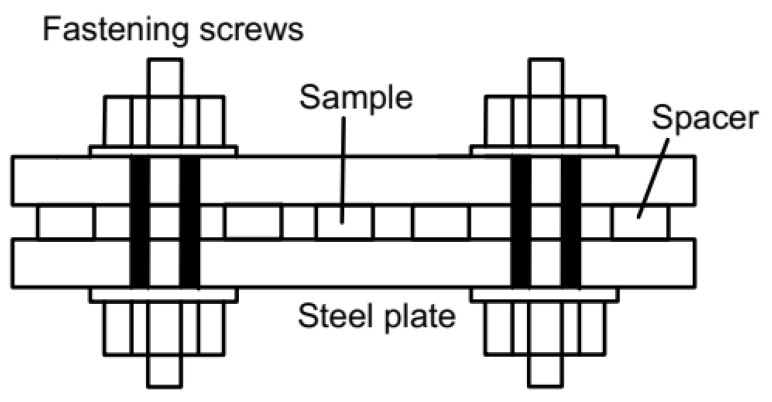
The diagram of compression apparatus.

In addition, the compression stress of the silicone rubber sample is applied by placing the silicone rubber O-ring in the compression simulators. It is composed of a steel plate, stopper, bolt, and nut. Silicone rubber samples and stoppers are placed in the middle of the steel plate, and the pressure is provided by fastening bolts and nuts. The height of the stopper is used to control the compression amount of silicone rubber samples, which is shown in Figure 2.

**Figure 3:**
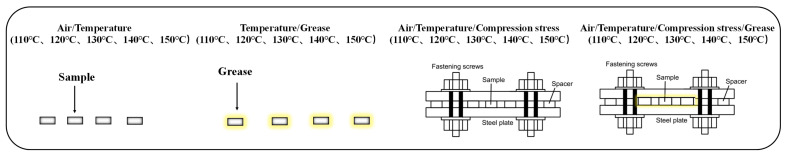
Schematic diagram of accelerated aging test scheme.

Finally, based on the above preliminary analysis and work preparation, we designed an accelerated aging test scheme plan for silicone rubber as shown in Figure 3. To ensure the reliability of test results, four parallel specimens were set under each aging condition and accelerated stress level, and the samples were examined after 2, 5, 8, 15, 27, 40, and 54 days, respectively. All samples will be cleaned with distilled water to remove residual oil from the surface in preparation for subsequent testing.

### 2.4. Characterization Methods

In this subsection, the aged silicone rubber samples were analyzed from the aspects of macroscopic physical properties and the microstructure, and the corresponding characterization methods were proposed.

#### 2.4.1. Physical Property Analysis

- Weight Loss
  The weight of the samples before and after aging under different conditions were measured through electronic balance, which has a resolution of 0.001 g. The percent weight loss (WL) was calculated from the following equation:
  (1) $$\mathrm{WL}=\frac{M_{1}-M_{2}}{M_{1}}\times 100\%,$$
  where $M_{1}$ is the initial weight of the sample, and $M_{2}$ is the final weight. All samples were measured after being placed under standard laboratory conditions for 24 h, and the final result was the average of four parallel samples.
- Compression Set
  The compression set (CS) reflects the ability of rubber to resist deformation under constant compression [28,29]. The samples after aging should be placed in a laboratory standard environment for 24 h after removal. Then, the CS of samples was calculated via the following equation:
  (2) $$\mathrm{CS}=\frac{H_{0}-H_{2}}{H_{0}-H_{1}}\times 100\%,$$
  where $H_{0}$ is the initial sample height, $H_{1}$ is the compressed height, and $H_{2}$ is the height at which the final sample no longer deforms. All results were averaged over four parallel samples.

#### 2.4.2. Microstructure Analysis

- ATR-FTIR analysis
  Attenuated total reflection-Fourier transform infrared (ATR-FTIR, Thermo Nicolet 6700) spectroscopy is used to evaluate changes in the chemical structure of the surfaces of seals under different states. The scanning spectrum ranges from 400 to 4000 cm${}^{-1}$, the resolution is 0.4 cm${}^{-1}$, and the wave number accuracy is 0.01 cm${}^{-1}$.
- X-ray photoelectron spectroscopy (XPS) analysis
  The role of XPS is to detect the changes in the types and relative contents of elements on the surface of samples before and after aging. The test temperature range of the equipment is 25–400 ${}^{\circ }$C.
- Thermogravimetric analysis (TGA)
  The TGA is utilized to study the change of thermal stability of silicone rubber before and after aging [30,31]. The test conditions were the thermal degradation of silicone rubber samples in an air atmosphere, the temperature range was 25–800 ${}^{\circ }$C, and the heating rate was 10 ${}^{\circ }$C/min.
- Scanning Electron Microscope (SEM) analysis
  In this paper, the SEM (SUPRA55, Beijing, China, Voltage 3 kV) was utilized to analyze the changes in the surface morphology of silicone rubber samples before and after aging under different working conditions.

## 3. Results and Discussion

In this part, the data and test results under various working conditions will be given, and the aging behavior and degradation mechanism of silicone rubber will be analyzed on this basis.

### 3.1. Weight Loss

**Figure 4:**
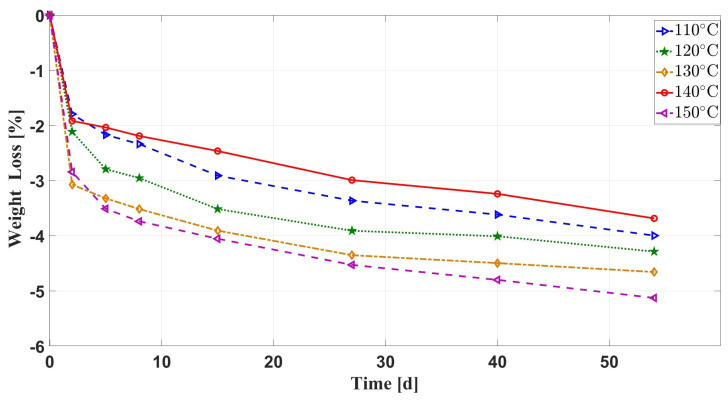
Comparison of weight loss of silicone rubber samples at different temperatures under multiple environmental aging conditions with aging time.

Figure 4 demonstrates the variation of the weight loss rate of silicone rubber with aging time at different aging temperatures under the effects of multiple environmental factors. It can be seen that the curves have roughly the same trend and are all negative, which indicates that the weight of the specimen is increased during the aging process under the action of multiple factors, such as air, compressive stress, oil, and temperature. The weight of the silicone rubber specimen increases rapidly during the pre-aging period (0–2 days), and the rate of its weight increase slows down after 2 days. When the aging temperature is 110–130 ${}^{\circ }$C, the weight increases with the increase in temperature. At 140 ${}^{\circ }$C, the weight of the silicone rubber specimen increases the least and at 150 ${}^{\circ }$C, the weight increases the most.

During the degradation process of silicone rubber under the synergistic effects of the above four environmental factors, there are a variety of reasons that can cause changes in its weight. Firstly, water and additives in silicone rubber specimens can escape in large quantities, which can lead to a reduction in specimen weight. According to the references [32,33,34,35], high temperature promotes the escape process, while oil and compression stress inhibit it. Secondly, small molecules of oxygen and oil can enter the silicone rubber matrix by diffusion, leading to a rise in weight. As can be seen from the previous section, high temperature has a positive effect on the diffusion of small molecules, and the oil has an inhibiting effect on the diffusion of small molecules of oxygen into the silicone rubber matrix, but the compressive stress at the same time will result in the silicone rubber molecular-chain-breakage molecular-chain-stacking rearrangement. Under the action of high-temperature, the silicone rubber molecular chain movement ability increases, making the silicone rubber matrix easily form connected cavities, which is conducive to the diffusion of small molecules in the silicone rubber matrix of the larger volume of the oil liquid. Therefore, through the above analysis, it can be seen that several factors work together and compete with each other, resulting in different changes in the weight of silicone rubber under the synergistic effect of multiple environmental factors.

In the early stages of aging (0–2 days), the rapid increase in sample weight is primarily driven by the diffusion of small oil molecules into the silicone rubber matrix. Moreover, at temperatures between 110 ${}^{\circ }$C and 130 ${}^{\circ }$C, the higher the temperature, the more intense the diffusion of oil molecules, resulting in a greater increase in the weight of the silicone rubber samples. When the aging temperature is raised to 140 ${}^{\circ }$C, moisture, additives, and oxidation products within the silicone rubber start to escape in large quantities, leading to a decrease in the mass of the silicone rubber. This is why the weight increase rate is at its minimum at 140 ${}^{\circ }$C. However, when the aging temperature is further increased to 150 ${}^{\circ }$C, the elevated temperature enhances the mobility of silicone rubber polymer chains, leading to the formation of interconnected, larger voids within the silicone rubber matrix. This, in turn, allows a large volume of oil molecules to diffuse once again into the silicone rubber matrix, resulting in an increase in the weight of the silicone rubber samples.

### 3.2. Compression Set

**Figure 5:**
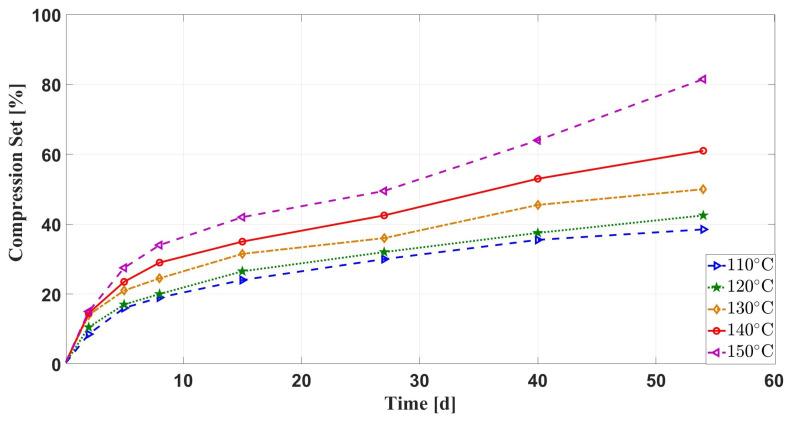
Comparison of compression set of samples with aging time under multiple environmental factors.

**Figure 6:**
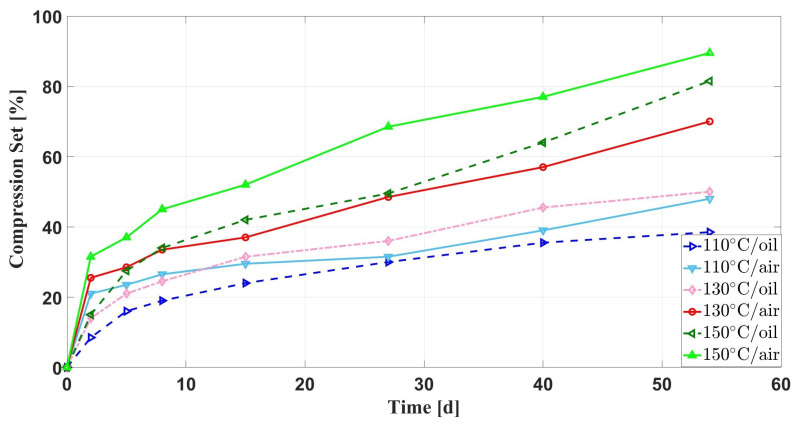
Comparison of compression set of samples with aging time under different aging conditions.

Figure 5 shows the variation curves of the compression set with the aging time of silicone rubber specimens under the effect of multiple environmental factors. It can be seen that the higher temperature, the greater compression set rate of the silicone rubber specimens. At 110–140 ${}^{\circ }$C, the changing trend of the compression set rate of the silicone rubber samples is roughly the same, mainly rapidly increased in the first two days of aging, decreased in the middle and late aging stages, and the compression set rate and aging time have an almost linear relationship. When the aging temperature is 150 ${}^{\circ }$C, the rate of increase in the compression set of the silicone rubber specimens in the late aging stage is obvious. Additionally, Figure 6 shows the curve comparison of the compressive set rate of silicone rubber samples under thermal stress and multiple environmental factors against the aging time. It can be seen that the compressive set rate of the latter increases more slowly under the same aging temperature.

Based on the above description of the aging phenomenon, we can give the following explanation. Firstly, under the joint action of multiple environmental factors, the rapid increase in the compression set rate in the pre-aging period is mainly because the oil-soluble additives in the silicone rubber matrix are extracted by the oil and volatilized under the action of high temperature, which leads to a decrease in the mechanical properties of the silicone rubber and an increase in its compression set rate. Secondly, in the middle and late stages of aging, the compression set rate increases almost linearly with the aging time, which is mainly due to the diffusion of air into the silicone rubber matrix and oxidation reaction with its molecular chains, leading to cross-linking, resulting in the compression set rate of the silicone rubber sample. Finally, at the late stage of aging at an aging temperature of 150 ${}^{\circ }$C, the rate of compression set increases dramatically, which may be due to a change in the aging mechanism with increasing temperature. It can be seen from Figure 6 that the compression set rate with oil is small and the increase rate is moderate in the early aging period. This is because the oil diffused into the silicone rubber matrix causes swelling, which has a certain counteracting effect on the compression stress, and the oil has an isolated effect on the air, slowing down the cross linking caused by the oxidation reaction between oxygen and the silicone rubber molecular chain.

### 3.3. ATR-FITR Analysis

**Figure 7:**
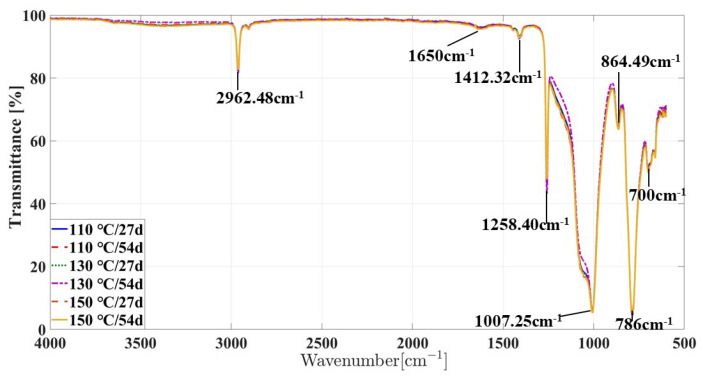
ATR-FTIR spectra of silicone rubber as a function of aging time at different aging temperatures under multiple environmental factors.

In this section, the ATR-FITR was performed on the aged specimens of silicone rubber via multiple environment factors with aging temperatures of 110 ${}^{\circ }$C, 130 ${}^{\circ }$C, 150 ${}^{\circ }$C and aging times of 27 days and 54 days, respectively, as shown in Figure 7.

As can be seen from it, compared with the infrared spectrum of non-aging silicone rubber, under the action of multi-factor coupling, the infrared spectrum of silicone rubber samples with different temperatures and aging time has a new absorption peak near the wave number 1650 cm${}^{-1}$, corresponding to the stretching vibration of the functional group C=O. The oxygen diffused into the silicone rubber matrix and the silicone rubber molecular chain oxidized to produce a small amount of oxidation products.

Under the action of multiple environmental factors, the absorption peak intensity corresponding to different wave numbers of the samples with different aging temperatures and time is not significantly different in the infrared spectral diagram, which indicates that under the action of multi-factor coupling, the aging degree of the silicone rubber samples is relatively stable within the aging time range, and there is almost no obvious aging with the increase in the aging temperature and aging time.

**Figure 8:**
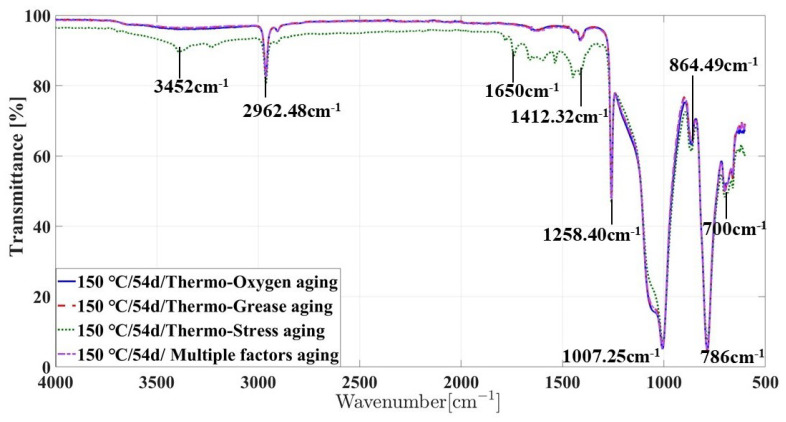
ATR-FTIR spectra of silicone rubber as a function of 54 days at different aging conditions.

The infrared spectra of silicone rubber samples under different aging conditions were compared and analyzed, as shown in Figure 8. It was found that under the same aging temperature and aging time, the infrared spectra of silicone rubber samples with hot oxygen aging, hot oil aging, and multi-factor coupling aging were roughly the same, and there was no obvious difference. There are new absorption peaks near the wave number 1650 cm${}^{-1}$, which indicates that there is the formation of the oxidation product C=O functional group during the aging process. Under the condition of thermal stress, not only does the absorption peak representing the functional group of oxidation product C=O appear but also the absorption peak representing hydroxyl (-OH) appears, and the corresponding absorption peak intensity of the two oxidation products is the largest in all aging conditions.

According to the above analysis, the aging degree of silicone rubber is the largest under the condition of thermal stress. Under the conditions of thermal oxygen aging, thermal oil aging, and multi-factor coupling aging, the aging degree is roughly the same, which indicates that compressive stress can promote the aging of silicone rubber. The lubricating oil has a very obvious protective effect on the silicone rubber under compression, which can greatly delay the aging of the silicone rubber seals that are often subjected to compression stress in the actual service environment.

In order to describe the microscopic chemical reactions and generated products during the aging process of silicone rubber as much as possible, we listed the following Table 3.

### 3.4. XPS Analysis

**Figure 9:**
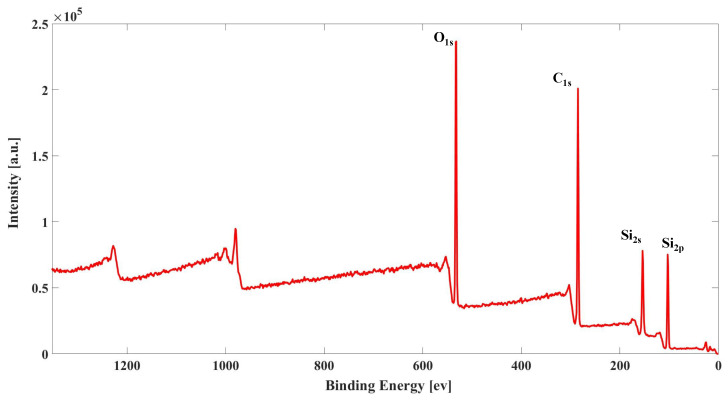
Total XPS spectrogram of the unaged silicone rubber sample.

Figure 9 shows the XPS scanning full spectrum of the unaged rubber sample, from which it can be seen that the main elements on the surface of silicone rubber are O, C and Si.

Under the combined action of four environmental factors, XPS full spectrum scanning was performed on silicone rubber samples with aging temperatures of 130 ${}^{\circ }$C, 150 ${}^{\circ }$C, and 27 and 54 days respectively. The results showed that the main elements on the surface of the samples did not change before and after aging, and were still O, C, and Si elements. The relative contents of each element are shown in Table 4.

It can be observed that among the three elements, the relative content of O increases the most at 130 ${}^{\circ }$C for 54 days, while the relative content of C decreases the most at 130 ${}^{\circ }$C for 27 days. In contrast, the Si element shows an initial increase in content with higher aging temperatures and times, followed by a decrease. Furthermore, under the same temperature conditions, an increase in the aging time leads to a slight rise in the content of O and C elements, while the Si element content decreases.

From the above results and combined with ATR-FITR, when the aging temperature is 130 ${}^{\circ }$C, oxygen diffuses into the silicone rubber matrix, and there is an oxidation reaction with the silicone rubber molecular chain side chain methyl, the oxidation product functional group (C=O) is formed, so the relative content of the O element increases. However, the surface of the silicone rubber sample in contact with the compression device only has a very small amount of grease attached due to the extrusion action, so the relative content of the C element on the surface of the sample does not increase significantly. With the aging process, the oxidation products continue to increase, and due to the effect of compressive stress, the silicone rubber network structure is destroyed, and the relative content of the O element increases, and the relative content of the Si element decreases when aging for 54 days. When the aging temperature is 150 ${}^{\circ }$C, the relative contents of major elements on the surface of the sample aged for 27 days are roughly the same as those on the surface of the sample aged for 54 days at 130 ${}^{\circ }$C, indicating that the degree of aging of the sample under these two aging conditions is consistent. The microscopic changes are the formation of some oxidation products C=O and the destruction of the network structure on the surface of the silicone rubber sample. When aging to 54 days, the relative contents of O and Si elements on the surface of the sample are decreased, while the relative contents of C elements are increased, which may be because the high temperature promoted the diffusion of small oil molecules towards the extruded surface, and the compression stress caused the formation of larger volumes of connected holes in the shallow matrix of the extruded surface of the silicone rubber. The small molecules of the oil are diffused into the shallow layer of the silicone rubber surface, resulting in a substantial increase in the relative content of the C element and a decrease in the relative content of the other two elements.

In summary, under the synergistic effects of the above environmental factors, the molecular structure of silicone rubber mainly occurs through the oxidation reaction of the molecular chain side chain, the destruction and cross linking of the silicone rubber network structure, and the diffusion of small oil molecules to the shallow layer of silicone rubber pressure surface under the action of compression stress.

### 3.5. Thermogravimetric Analysis

Under the synergistic effects of multiple environmental factors, thermal analysis was performed on silicone rubber samples at different aging temperatures (110 ${}^{\circ }$C, 130 ${}^{\circ }$C, 150 ${}^{\circ }$C) and times (54 days), and the key data are shown in the following Table 5. In this table, “DIT” represents “Decomposition initiation temperature”, and “FRD” represents the fastest decomposition rate point.

With the increase in temperature, the initial temperature of decomposition and the temperature point of the fastest decomposition rate decrease, which may be because the compressive stress destroys the network structure of silicone rubber. With the increase in temperature, the movement ability of the molecular chain of silicone rubber is intensified, and connected holes with large volumes are easily formed in the silicone rubber matrix. However, these holes will be occupied by small oil molecules, and the damaged silicone rubber network structure cannot form a cross-linking reaction, and the cross-linking degree decreases, resulting in a decrease in the decomposition starting temperature and the fastest decomposition rate temperature point with the increase in the aging temperature.

In addition, the comparative data of residual weight after thermal degradation of silicone rubber samples at an aging temperature of 150 ${}^{\circ }$C and aged for 54 days under different aging conditions are shown in Table 6. As can be seen from the above two tables, the residual weight of the samples after aging decreases compared with the samples without aging, and the higher the aging temperature, the smaller the residual mass. When the aging temperature is the same, the residual mass of the aging sample under multi-factor coupling is much smaller than that under other aging conditions, which may be due to the formation of larger connected cavities in the silicone rubber matrix under the action of compressive stress. The higher the temperature, the more that cavities are formed, and a large number of small oil molecules occupy these cavities, which also increases the weight of the aging sample. In the process of thermal degradation, due to the action of high temperature, a large number of small oil molecules escape from the cavity, resulting in a large reduction in the sample mass and a relatively small residual weight.

### 3.6. SEM Analysis

**Figure 10:**
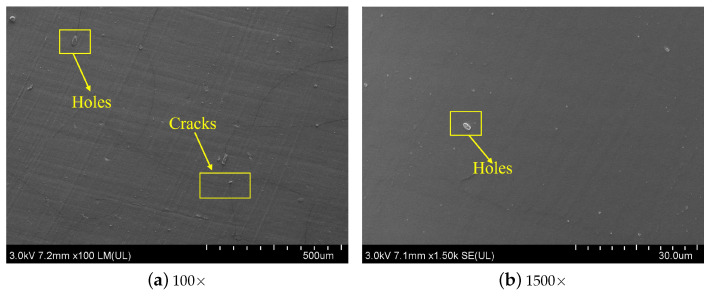
SEM of the surface morphology of silicone rubber in contact with compression device after 54 days of aging at 150 ${}^{\circ }$C under multiple environmental factors.

**Figure 11:**
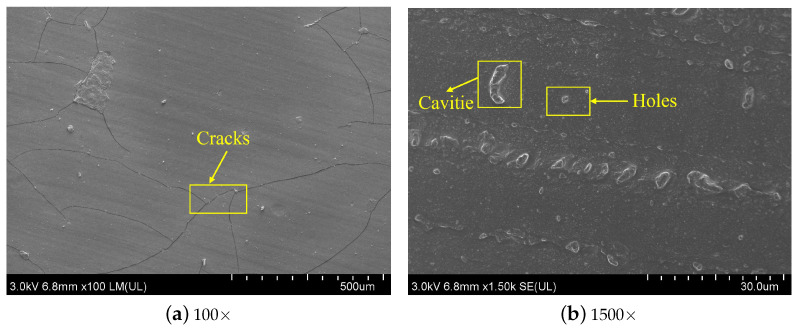
SEM of the surface morphology of silicone rubber in contact with air after 54 days of aging at 150 ${}^{\circ }$C under multiple environment factors.

In this section, SEM was fully utilized to observe the micromorphology of silicone rubber samples before and after aging. Figure 10 shows the morphology of the surface of the silicone rubber aged specimen in contact with the compression device. After an aging temperature of 150 ${}^{\circ }$C and aging for 54 days, there are obvious cracks on the surface under low magnification, and holes can be seen under high magnification. While Figure 11 demonstrates the morphology of the surface of the aged silicone rubber specimen in contact with the air under the same conditions, it can be seen under low magnification that there are obvious cracks on the surface, and under high magnification, it can be seen that there is the emergence of holes and cracks, and both of them are deeper than the surface that is in contact with the compression device in the above figure.

According to the above results, the aging degree of the surface in contact with the compression device is less than that of the surface in contact with the air. Compared with the surface morphology of silicone rubber under other aging conditions, it can be seen that under the synergistic effects of multiple environmental factors, cracks and holes appear on its surface mainly due to the diffusion of small oil molecules into the silicone rubber matrix, resulting in the swelling of silicone rubber, resulting in stress concentration on the silicone rubber surface, resulting in the above cracks and holes.

## 4. Silicone Rubber Degradation Mechanism

Based on the analysis of the physical performance degradation behavior and microstructure changes of silicone rubber in the previous section, we propose the degradation mechanism of silicone rubber in the marine atmospheric environment in this section.

Firstly, the diffusion of small molecules of oil into the silicone rubber matrix leads to a rapid increase in the weight of the silicone rubber, while the extraction of small molecules of oil from the additives in the silicone rubber matrix leads to a decrease in the weight of the silicone rubber specimen. When the aging temperature is 110–130 ${}^{\circ }$C, the diffusion of small oil molecules into the silicone rubber matrix plays a dominant role, and the higher the temperature, the more intense the diffusion degree of the small oil molecules, which also explains that the higher the temperature in this temperature range, the greater the increase in the weight of the silicone rubber samples. When the aging temperature is 140 ${}^{\circ }$C, the rate of water and additives escaping from the silicone rubber matrix is greater than the rate of small oil molecules diffusing into the silicone rubber matrix, and the weight of the silicone rubber sample increases the least. When the aging temperature is 150 ${}^{\circ }$C, combined with the XPS analysis results, the compression stress destroys the structure of the rubber network, causing the molecular chains to be stacked and rearranged, while the high temperature enhances the movement ability of the silicone rubber molecular chains. Under the combined action of high temperature and compression stress, a large number of connected cavities are formed in the silicone rubber matrix. The small oil molecules with relatively large molecular weight and volume can be diffused into the silicone rubber matrix, which also explains that the weight of the silicone rubber sample increases the most at 150 ${}^{\circ }$C.

Based on the microstructure analysis results of the silicone rubber sample, the diffusion of small molecules in the oil into the silicone rubber matrix causes the swelling of the silicone rubber, resulting in stress concentration on the surface of the silicone rubber, and additives in the silicone rubber matrix are extracted and escaped by small molecules in the oil, all of which result in varying degrees of defects on the surface of the silicone rubber sample. Specifically, the surface sample in contact with the air has serious defects and obvious cracks and holes, while the sample on the contact surface with the compression device has a slight defect degree, which indicates that the aging of the silicone rubber sample under the action of various environmental factors is uneven. In addition, according to the results of ATR-FITR, with the aging process, small oxygen molecules diffuse into the silicone rubber matrix through the surface defects of silicone rubber and oxidize the side chain of the silicone rubber molecular chain. The generated oxidation product C=O functional group increases the diffusion coefficient of the small oil molecules, which also explains that the weight of the silicone rubber samples is increased with the aging time.

Secondly, due to the extraction effect of small oil molecules on silicone rubber additives, the mechanical properties of silicone rubber samples are decreased, which is most directly reflected in the increase in its compression set rate. According to the results of XPS and TGA, it can be seen that under the combined action of high temperature and compressive stress, hydrogen atoms and polar groups of oxidation products on C-H on the side chain of the silicone rubber molecular chain are easily broken from the molecular chain, destroying the silicone rubber network structure. The interaction between the generated free radicals will cause the cross-linking reaction of silicone rubber. Within a certain range, increasing the temperature can promote the progress of the cross-linking reaction, enhancing the cross-linking density and degree. However, excessively high temperatures may lead to polymer degradation or the occurrence of side reactions, thus affecting the effectiveness of the cross-linking reaction. It is also shows that with the aging process and the deepening of the cross-linking degree, it is difficult for the silicone rubber sample to recover from the deformation, that is, its compression set rate continues to increase with the aging temperature and time.

**Figure 12:**
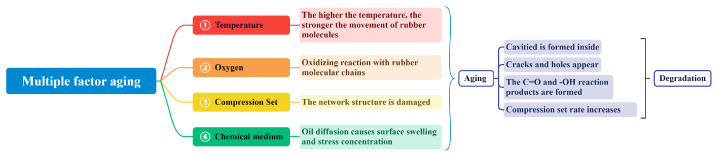
Schematic diagram of silicone rubber degradation mechanism under marine atmospheric environment.

For better comprehension, Figure 12 is a simplified schematic structural diagram illustrating the degradation mechanism of silicone rubber in a marine atmospheric environment.

## 5. Conclusions

In this paper, through the laboratory simulation of a marine atmospheric environment, an accelerated test was designed, and the degradation behavior and failure mechanism of silicone rubber seals under the synergistic effects of multiple environmental factors (air, temperature, compression stress, and chemical media) were systematically studied from both macro and micro aspects. In terms of mechanical properties, the weight of the silicone rubber samples shows an increasing trend during the whole aging process, but it does not mean that the higher the temperature, the greater the weight increase, which indicates that the weight change is the result of multiple environmental factors working together and competing with each other. The results show that oil is the most important environmental factor affecting the weight of the silicone rubber samples, followed by temperature and compressive stress. Meanwhile, for the compression set rate, the rapid increase in the early stage is mainly due to the oil-soluble additives in the silicone rubber matrix being extracted by the oil and volatilized at high temperatures, resulting in the decline of its mechanical properties. The compression set rate increases linearly with the aging time in the middle and late stages, mainly because the air diffuses into the matrix and oxidizes the silicone rubber molecular chain, leading to cross linking. Additionally, combined with micro-analysis methods such as ATR-FITR, XPS, and TGA, the micro-molecular structure of silicone rubber under multiple environmental factors is mainly manifested as the formation of a small amount of the oxidation product C=O functional group, the destruction of the silicone rubber network structure, the cross linking between silicone rubber molecular chains, and the diffusion of small oil molecules to the shallow layer of the silicone rubber under compression stress. Due to the diffusion of small oil molecules into the silicone rubber matrix, a large number of cracks and holes on the surface of silicone rubber can be seen under SEM. Therefore, based on the above results and analysis, the aging of silicone rubber under multiple environmental factors is uneven.

The future work can be carried out in the following two aspects. Firstly, facing the specific marine environment, the aging behavior and failure mechanism of silicone rubber seals under harsh environments, such as strong ultraviolet, high humidity, and salt spray, can be well considered. Secondly, the data obtained in the accelerated test can be utilized to predict the life of the silicone rubber sealing ring sample by establishing appropriate mathematical models.

## Figures and Tables

**Table 1:** Constituents of silicone rubber samples.

Constituents	wt%
Silicone rubber	62.3
Fumed silica	23.6
Hydroxyl silicone oil	7.8
Zinc oxide	3.2
Ferric oxide	1.8
2,5-Dimethyl-2,5-di(tert-butylperoxy) hexane	1.2

**Table 2:** The main parameters of the 7017-1 grease.

Test Item	Measured Parameter
Appearance	Gray uniform ointment
Dropping point	318
Penetration	72
Evaporability	0.75
Pressure oil separation	7.34
Similar viscosity	1492

**Table 3:** The chemical reaction process of silicone rubber.

Chemical Reaction Process in the Aging Process of Silicone Rubber
**Step 1** **Initial siloxane chain structure**
Siloxane chain (-Si O-Si O-Si-...).
**Step 2** **Oxygen action**
Oxygen molecules ($O_{2}$) will react with silicon (Si) atoms in the siloxane chain.
**Step 3** **Oxidation of siloxane chains**
The oxidation reaction between oxygen and silicon atoms in the siloxane chain
leads to the breaking of silicon oxygen (Si-O) bonds and the insertion of oxygen atoms.
**Step 4** **Generation of Silanol (Si-OH) functional groups**
Some silicon atoms on the siloxane chain will react with oxygen to form silanol functional
groups (Si-OH), where -OH represents hydroxyl groups.
**Step 5** **Continue oxidation**
The silanol functional group can continue to oxidize, and oxygen further reacts with it,
causing the oxygen atoms in the silanol functional group to form a C=O functional group
with silicon, which is the formation of carbon oxygen double bonds (C=O).
**Step 6** **C=O functional group formation**
Ultimately, the oxidation reaction in the silanol functional group leads to the formation
of the C=O functional group, i.e., the carbon oxygen double bond C=O.

**Table 4:** Relative contents of main elements on silicone rubber surface at different aging temperatures and time under multiple environmental factors.

Test Conditions	Relative Content		
	O	C	Si
Unaged sample	23.35%	51.9%	24.75%
130 ${}^{\circ }$C/27 d	24.76%	49.31%	25.93%
130 ${}^{\circ }$C/54 d	25.26%	50.6%	24.12%
150 ${}^{\circ }$C/27 d	25.13%	50.44%	24.43%
150 ${}^{\circ }$C/54 d	22.82%	54.5%	22.69%

**Table 5:** Thermal analysis data of silicone rubber samples at different aging temperatures under multiple environmental factors.

Test Conditions	DIT	FRD	Residual Weight
Unaged sample	376 ${}^{\circ }$C	425.7 ${}^{\circ }$C	54.5%
110 ${}^{\circ }$C	437 ${}^{\circ }$C	501.4 ${}^{\circ }$C	39.29%
130 ${}^{\circ }$C	408 ${}^{\circ }$C	493.7 ${}^{\circ }$C	37.91%
150 ${}^{\circ }$C	403 ${}^{\circ }$C	476.1 ${}^{\circ }$C	26.16%

**Table 6:** Comparison of residual weight of silicone rubber samples under different aging conditions.

Sample Conditions	Residual Weight
Unaged sample	54.5%
150 ${}^{\circ }$C, 54 d/Thermo-Oxygen Aging	36.43%
150 ${}^{\circ }$C, 54 d/Thermo-oil Aging	41.42%
150 ${}^{\circ }$C, 54 d/Thermo-stress Aging	44.67%
150 ${}^{\circ }$C, 54 d/Multiple environmental factors Aging	26.16%

## Data Availability

Not applicable.
